# Editorial: Optimizing the first-line treatment for metastatic colorectal cancer

**DOI:** 10.3389/fonc.2024.1366383

**Published:** 2024-01-24

**Authors:** Davide Ciardiello, Angelica Petrillo

**Affiliations:** ^1^ Division of Gastrointestinal Medical Oncology and Neuroendocrine Tumors, European Institute of Oncology, IEO, IRCCS, Milan, Italy; ^2^ Medical Oncology Unit, Ospedale del Mare, Naples, Italy

**Keywords:** CRC (colorectal cancer), anti-EGFR, panitumumab, QOL (quality of life), liver metastasis, biomarkers

The prognosis of metastatic CRC (mCRC) patients has significantly improved in the past 20 years thanks to the introduction of novel therapeutic approaches, including surgery of liver, lung and peritoneal metastases, and of more effective anticancer drugs and combinatory strategies (1). As the maximum clinical benefit is achieved during first-line treatment, the choice of the best front-line treatment represents the keystone in the whole therapeutic strategy. Current treatment options include the combinations of chemotherapy agents such as 5-Fluorouracil, oxaliplatin and/or irinotecan with epidermal growth factor receptor (EGFR) inhibitors in RAS/BRAF wild-type patients or with the anti-angiogenic drugs bevacizumab. Additionally, the introduction of immune checkpoint inhibitors has revolutionized the treatment of patients with microsatellite unstable CRC.

Alongside the increasing number of therapeutic options, there are still different matters of debate, including the treatment of frail patients (un-fit for intense chemotherapy), the duration of induction strategy/perioperative treatment, the right timing of surgery and loco-regional treatment in the therapeutic program, the use of the maintenance therapy, the possibility of “therapeutic holidays” in selected patients and the identification of novel biomarkers and prognostic score to optimize the available treatment for each patient.

This Research Topic entitled “*Optimizing the First-Line Treatment for Metastatic Colorectal Cancer*” aimed to collect relevant original research (clinical and translational), high-quality reviews, meta-analyses and short communications covering the above-mentioned.

Overall, 14 manuscripts were submitted, of these 6 (2 reviews and 4 original articles) were accepted for publication (acceptance rate 43%) with a total of 44 authors and 13 reviewers involved. In this editorial, we summarize some of the main topics that were faced in the Research Topic.

So far, the decision on the best first-line treatment has a crucial role in the whole therapeutic journey of a patient with mCRC since it has the highest possibility to provide a tumor response and influence the subsequent/s therapies. In this scenario, Cherri et al. did an elegant overview of the current challenges and future perspectives in the treatment of unresectable mCRC. The authors turn on the lights on the molecular landscape of mCRC and propose a therapeutic algorithm for each subgroup.

Considering the improvement in the overall survival (OS) of patients with mCRC over time, the study and preservation of quality of life (QoL) is becoming a priority in this field. Nevertheless, QoL is evaluated mostly in a later-line setting, whereas it is frequently underestimated in the first-line. Castells et al. provide a comprehensive evaluation of the emerging role of the patient-reported outcome (PRO), patient advocates and QoL in the implementation of clinical trials and daily practice.

The combination of capecitabine with irinotecan and the anti-EGFR panitumumab (CAPIRI-P) represents a controversial scheme to use in mCRC due to the cumulative gastrointestinal toxicity. Nevertheless, the safety profile could be improved by modifying and reducing the dosage of the drugs. In this Research Topic, Yip et al. evaluated the feasibility of modified CAPIRI-P in a real-world cohort of 106 Asian patients with mCRC. Of note, the median progression-free survival (mPFS) and mOS were 15.4 (95% confidence interval (CI) 12.5–18.3) and 25.5 (95% CI 17.6–33.4) months, respectively. Grade ≥3 toxicity rate was 43%, predominantly related to uncomplicated hematological toxicities.

Still about chemotherapy choice in the first-line setting, in the Research Topic Song et al. showed the preliminary results of a single-arm phase II trial that investigated the combination of the CAPOX regimen with anlotinib in 31 patients with mCRC. No signals of unexpected toxicities were reported; however, despite the signals of activity, the study did not meet the primary endpoint and was not able to demonstrate a significant benefit in terms of PFS compared with historical control. A longer follow-up for survival and biomarker analyses is required before moving to a subsequent step of clinical development with a randomized study.

Regarding the metastatic sites in the case of mCRC, liver is the most frequent with around 2 out of 3 patients interested (2). Thus, a multimodality approach with liver locoregional treatment and systemic therapies could have a curative intent in a subgroup of selected mCRC patients. In this Research Topic, Yu et al. investigated the effect of microwave ablation (MWA) on the immune system in a pre-clinical model. CT26 tumor-bearing Balb/c mice were treated with MWA evaluating different combinations of ablation power and treatment duration. Interestingly, MWA induced both local and systemic T-cell responses. Additionally, the mice displayed a significant abscopal effect after MWA with an increase in Th1 cell proportion both in the tumors and spleens.

Finally, Chen et al. assessed different prognostic factors in a population of 85 mCRC patients who received surgical resection of colorectal cancer liver metastases (CRLM). Of note, the microsatellite stable (MSS) status, high Ki-67, and lymph-vascular invasion were independent risk factors for worse survival. These results might be taken into account, but further prospective evaluation is needed to better define tumors at increased risk of recurrence following radical resection.

In conclusion, in the era of precision medicine and multidisciplinary approaches, the therapeutic armamentarium for mCRC has rapidly evolved over time (3). Consequently, the complexity of the treatment decision is a major challenge for clinicians in the daily clinical practice; it should be taken after multidisciplinary discussion. Thus, the main goal is to implement the efficacy of the individualized treatment without forgetting the cost in terms of QoL (4). In this scenario, further translational studies are required to identify potential biomarkers to personalize the available therapeutic strategies, define a tailored continuum of care and design innovative clinical trials.

## Author contributions

DC: Conceptualization, Writing – original draft, Writing – review & editing. AP: Conceptualization, Writing – original draft, Writing – review & editing.
